# Neuropsychological and Psychological Functioning Aspects in Myotonic Dystrophy Type 1 Patients in Italy

**DOI:** 10.3389/fneur.2018.00751

**Published:** 2018-09-19

**Authors:** Edward Callus, Enrico G. Bertoldo, Maria Beretta, Sara Boveri, Rosanna Cardani, Barbara Fossati, Elisa Brigonzi, Giovanni Meola

**Affiliations:** ^1^Clinical Psychology Service, IRCCS Policlinico San Donato, San Donato Milanese, Italy; ^2^Scientific Directorate, IRCCS Policlinico San Donato, San Donato Milanese, Italy; ^3^Laboratory of Muscle Histopathology and Molecular Biology, IRCCS Policlinico San Donato, San Donato Milanese, Italy; ^4^Department of Neurology, IRCCS Policlinico San Donato, San Donato Milanese, Italy; ^5^Department of Biomedical Sciences for Health, University of Milan, Milan, Italy

**Keywords:** myotonic dystrophy, neuropsychological assessment, psychological assessment, neuropsychological functioning, psychological funtioning, patient empowerment

## Abstract

**Introduction:** Myotonic Dystrophy Type 1 (DM1) is an autosomal dominant genetic illness, characterized by a progressive loss of strength. Important deficits in cognitive functioning and a significant prevalence of psychiatric disorders have been previously reported.

**Methods:**A neuropsychological and psychological assessment was carried out in 31 DM1 patients (61% males) in order to measure the cognitive functioning and explore their personality profiles. The MMSE Mini-Mental State Examination, Frontal Assessment Battery (FAB), ENB-2 Battery assessing memory (short term, long term and working memory), integration capacities, visual-spatial ability, attention (selective, divided, shifting/switching) executive functions, praxis, discrimination and logic capabilities and psychopathology Symptom Check List 90-R (SCL-90-R) were administered. The neuropsychological and psychological evaluation of DM1 patients was carried out taking into consideration the clinical parameters (CTG repeat, age at onset, disease duration, Muscular Impairment Rate Scale (MIRS), Medical Research Council Scale (MRC) and the Epworth Sleepiness Scales (EPS)).

**Results:** Regarding psychopathology 19.4% of patients scored a moderate or high level of symptoms intensity index (GSI), 12.9% reported a high number of symptoms (PST) and 16.1% reported a high intensity level of the perceived symptoms (PSDI). Fatigue and daytime sleepiness resulted as being associated with higher levels of psychoticism (PSY). Only 1 patient reported a severe impairment in the spatial and temporal orientation, memory, language, praxis, attention and calculation. Longer disease duration was also associated with cognitive impairment evaluated through ENB-2 (*p* < 0.05).

**Discussions and Conclusions:**There are indications of the utility of neuropsychological and psychological screening and support for these patients and their families due to the link between disease duration and cognitive performances. A proposal of a clinical protocol, with an illustration of a clinical case report of a family is presented.

## Introduction

Myotonic dystrophy type 1 (DM1; OMIM #160900) is a multisystemic neuromuscular disorder, which represents the most common form of muscular dystrophy in adults (1). DM1 is an autosomal dominant disease caused by an expansion of unstable CTG repeats located within the 3′-UTR of the *dystrophia myotonica protein kinase* (DMPK) gene. Mutant transcripts contain the triplet repeats form RNA hairpins that accumulate as foci in cell nuclei (2–4). These toxic transcripts are thought to sequester alternative splicing regulators leading to splicing defects that are considered the primary cause of DM1 symptoms (5). DM1 patients suffer from muscle wasting, weakness, and myotonia but also from heart conductions defects and central nervous system alterations (1). An important characteristic of DM1 that can influence adults and children at birth (congenital DM1, CDM) or during childhood is anticipation, with the aggravation of the disease severity and onset at an earlier age through successive generations. Five main categories in which DM1 patients can be divided into have been identified by the IDMC—International Myotonic Dystrophy Consortium (6); “congenital, childhood-onset, juvenile, adult-onset, and late onset/asymptomatic.” In each one of these five forms, specific clinical features and management problems are described, and there is a distinction from each other based on the prevalence of the main symptoms and apparition profiles.

It is well-known that brain involvement results in cognitive and psychiatric dysfunction, which have an important impact on patient quality of life (QoL) (7–9). Neurocognitive dysfunction such as intellectual disabilities, reduced IQ-values, speech and language delay, deficit in visuospatial and/or visuo-constructive skills is a hallmark of the childhood forms of DM1 (infantile and juvenile onset forms) (8, 10–14). This dysfunction is comprehensive of neuropsychiatric problems, such as predominantly inactive subtype of attention deficit hyperactive disorders or autism spectrum disorders (11, 12). The juvenile form is characterized by school and problems with peers and is often under-recognized. These childhood forms of DM1 have to be considered as a central nervous system (CNS) disease rather than a muscular or systemic disease (15). In the adult-onset form of DM1, several studies have described selective impairments in executive function, visuospatial function, processing speed, and attention (8, 16–18). Characteristic personality patterns and emotional disturbances including fatigue, daytime sleepiness, depression, and apathy have also been reported in DM1 patients (19–21). DM1 patients have a clear age-related decline of cognitive functions as demonstrated through detailed neuropsychological studies, linguistic levels, praxis evaluations, and executive task evaluations. Two recent longitudinal studies have reported a cognitive decline especially for verbal memory, attention, visuo-spatial construction, and processing speed (22, 23) reinforcing the previous observation of Sansone et al. (17) who observed a progression of frontal cognitive impairment. The level of decline does not tend to correlate with either the number of CTG repeats or the severity of muscle weakness (16, 22, 23). These studies give support for proposals on a possible degenerative brain process (24). Recently, a review on central nervous system involvement, highlighted the wide phenotype and variable cognitive involvement that may occur in myotonic dystrophies (25).

In a recent study aimed to describe the psychological characteristics of a large cohort of patients with DM1 in Canada, it has been reported that DM1 patients are at high risk of developing a psychiatric disorder (26). Moreover, psychological traits differ across phenotypes, with the most severe phenotype tending to show more severe psychological symptoms. High levels of anxiety and low esteem are associated with lower levels of education and higher repetition of CTG (26). The literature about DM1 patient population highlights the predominance of paranoid together with aggressive traits (27). Furthermore, a clinically significant personality impairment, with dependent and paranoid personality patterns has been found and this, in turn, may decrease QoL (28).

Advanced MRI studies demonstrated across the brain a widespread white matter disruption and a multifocal gray matter volume loss by using various single MRI techniques, including diffusion tensor imaging and voxel-based morphometry, with correlations found between corresponding quantitative MRI parameters and triplet expansion, neuropsychological tests, and the severity of muscular involvement (24, 29–37). Abnormal patterns of brain connectivity have also been reported in DM1 patients and have been demonstrated to account for patients' personality traits (38–40). A study of neuroimaging of brain in children and adolescents suggests a relationship between white matter damage and working memory (34).

Currently there is still no comprehension regarding the links existing between pathomolecular mechanisms, genetic abnormalities and CNS involvement. It is vital to identify the adequate biomarkers of brain involvement in DM1 as CNS outcome measures are needed for upcoming gene therapy clinical trials (19, 25, 41, 42).

To date there is no cure or specific treatment for DM1 and treatment is aimed at managing symptoms of the disease, such as Mexiletine for myotonia and Modafinil for excessive daytime sleepiness. However, new therapeutic approaches based on pathogenic mechanisms at RNA or DNA level may become feasible in the near future

Currently, the best that can be done for DM1 patients is to give them the best care and clinical follow up, merging it with psychological assistance and support in order to prevent alienation. It is well-established that chronic diseases present challenges to health-related quality of life, an individual's perceived physical and mental well-being. Rare diseases may also create additional threats to quality of life due to poor access to information, treatment and support, combined with high levels of stigma. Psychotherapy can help not only this kind of patients, but also their families, to learn new behaviors and ways of coping with the symptoms of the disease.

In 2012 the OPTIMISTIC study (Observational Prolonged Trial In Myotonic Dystrophy type 1 to Improve Quality of Life–Standards, a Target Identification Collaboration), a project founded by the European Commission, coordinated by the Netherlands and involving collaborative partners from France, Germany and United Kingdom, was launched (43).

It was a multi-center, randomized trial, designed to evaluate, as primary objective, the effect and the maintenance of a tailored behavioral change intervention comprising cognitive behavioral therapy including graded exercise training against standard patient management on participation for severe fatigue, as measured by the DM1-Active scale (Rasch-built measure of activity and participation for DM1 people). This innovative study involved over 200 patients across Europe and represented the first project extending psychotherapy to a large cohort of DM1 patients. The intervention also involved caregivers.

All the studies carried out until now, give a strong indication that in DM1 patients, cognitive impairments and psychological problems can affect their behaviors toward medical providers and their access to appropriate care, leading to worse QoL. Thus, it appears that DM1 patients could obtain benefits improving patient support, especially social and psychological support, and by improving communication with patients.

It has been observed in the medical settings that patients often require more information than they are given from health professionals, and they feel that they would like to be more involved in therapeutic procedure (44). In order for an effective involvement of patients in their care and their increased engagement, it is essential to enact strategies to improve their health literacy and put them in a condition that they feel they have control on their disease (45). For this to occur it is also important to consider the patient's preferences and needs in the medical decision-making process (46).

In fact, it is also important to take into consideration the mental dimensions, which make all patients unique, along with the assessment of their biological and clinical characteristics, in order to be able to tailor the interventions to them (47). The more there are efforts to create a patient profile which takes into consideration as many psychosocial elements as possible, the more it is possible to give a personalized approach in healthcare, improving and facilitating the communication between the physician and the patient (48).

In order to be fully effective, personalized medicine needs patients to be engaged and informed about their condition, who are also encouraged to discuss what their various treatment options are and their consequence (49).

The main objective of our study is to carry out a neuropsychological (through ENB-2 battery, MMSE, FAB) and psychological assessment (through SCL-90-R) of patients diagnosed with DM1 in order to measure the cognitive functioning and explore the personality profiles of these patients (50) in order to have a general picture of the cognitive and psychological functioning of Italian DM1 patients. Based on the outcomes, a secondary objective is to envisage a neuro-rehabilitation and psychological support program.

On the basis of the Outcomes Measures in Myotonic Dystrophy type 1 (OMMYD-1) for our study we used neuropsychological tests and self-report symptom questionnaires that found consensus among scientific community and measure the same abilities (19)^1^. Based on the Canadian experience (26), the Clinical Psychology Service of IRCCS Policlinico San Donato has set up an assessment protocol aimed to describe the neuropsychological and psychological characteristics of DM1 patients and to evaluate and create a neuro-cognitive rehabilitation and psychological support service for the patients in order to preserve the cognitive reserve. The protocol has been designed to be administered in a time frame compatible with patient hospitalization or with their outpatient access to our Neuromuscular Center.

Due to the limitations of the sampling size, in this study the correlations between cognitive/frontal functioning and demographic, medical or psychological symptoms will be explored. This data will be used to better tailor the psychological and neuropsychological program which was created for the patients and their families

The assessment protocol has been tested on a small cohort of Italian DM1 patients and the psychological support program has been tested on a family with the father and two sons diagnosed with DM1 and the mother not affected by DM1.

## Materials and methods

The authors analyzed the theoretical background in the current literature on Myotonic Dystrophy in order to identify the state of art of patients' neuropsychological and psychological characteristics and to design an efficient protocol to be used in this study.

We conducted a review of current literature using the methodology explained in Appendix 1. The aim was the analysis of the available neuropsychological and psychological researches on DM1 patients and consider a plot of studies, which are presented in Table 1.

**Table 1:** Review of neuropsychological and psychological literature on myotonic dystrophy.

**Article**	**Country**	**DM form**	**Sample**	**Investigation areas**	**Materials**
Angeard et al. (11)	France	Childhood form	24 DM1	Neurocognition in childhood	MIRS WAIS-III
Bajrami et al. (51)	Turkey	All forms	13 DM1	Cognitive functions and cerebral involvement	MRI scans MIRS MMSE (*Mini-Mental State Examination*) MoCA (*Montreal Cognitive Assessment*) EHI (*Edinburgh Handedness Inventory*) WAIS Buschke Selective Reminding test (neuropsychological) FAB (*Frontal Assessment Battery*)
Baldanzi et al. (52)	Italy	Adult-onset form	65 DM1, 26 healty controls	Disease's awarness	MIRS MRC (*Medical Research Council scale*) TIB (*Brief Intelligence Test*) BDI-II STAI-Y2 AES (*Apathy Evaluation Scale*) INQoL (*Individualized Neuromuscular Quality of Life Questionnaire*) Neuropsychological cognitive battery: Immediate and Delayed Recall (IR, DR) RAVLT IR, DR of Rey-Osterrieth Complex Figure (ROCF) Digit span and CBT (*Corsi's block test*) TMT A, B Stroop Test FAS FAB WSCT Raven's progressive matrices (PM47)
Bertrand et al. (26)	Canada	Mild and adult-onset	200 DM1 (152 adult-conset form, 48 mild-compromission/late-adult)	Psychological characteristic	SCL-90-R (*Symptom Check list-90-Revised*) MIRS (*Muscular Impairment Rating Scale*) NEO-FFI (*Revised NEO Personality Inventory*) Rosenberg scale (self-esteem) ASIQ (*Adult Suicidal Ideation Questionnaire*) WAIS-R (*Wechsler Adult Intelligence Scale-Revised*)
Bird et al. (53)	USA	Adult-onset form	29 DM1	Cognitive functions and psychological characteristics	WAIS Shipley-Hartford Scale [pure verbal misure of intellectual functions] MMPI (*Minnesota Multiphasic Personality Inventory*)
Bungener et al. (54)	France	All except for congenital form	15 DM1, 14 healty controls, 11 with FSHD	Psychopathology	Semistructured interview DSM-III MADRS (*Montgomery and Asberg*) HDRS (*Hamilton depressive scales*) Covi and Tyrer anxiety scales AT (*Abrams and Taylor scale for emotional blunting*) EHD (*depressive mood scale*) PAS (*the physical anhedonia scale*) SAS (*social anhedonia scale*)
Cabada et al. (55)	Spain	Adult-onset form	42 DM1, 42 healthy	Cerebral involvement, cognitive functions and psychopathological traits	Diffusion tensor imaging WML (White Matter Lesions) MRI Digit Span TMT-B WAIS-IV (Letters and numbers) Cubes of the Barcelona Test Revised CPT (Continuous Performance Test) Benton Visual Retention Test TAVEC (Verbal learning Spain Complutense Test) Interview for Deterioration in Daily Living Activities in Dementia Dysexecutive Questionnaire HARS BDI-II SCL-90-R
Caso et al. (30)	Serbia	Juvenile and adult-onset form	52 DM1, 34 controls	Cognitive impairment and cerebral damage	MRI scans ACE-R (*Addenbrooke's Cognitive Examination–Revised*) Raven progressive matrices WAIS Digit symbol coding Arithmetic subtests TMT WCST WAIS digit span ACE-R memory subscore RAVLT ROCF ACE-R (*Addenbooke's Cognitive Examination*) visuospatial subscore ROCF Copy Test WAIS block design VOT (*Hooper Visual Organization Test*) ACE-R language subscore BNT (*Boston Naming Test*)
Colombo et al. (56)	Italy	Severe form	40 DM, 20 healty controls	Neuropsychological and psychiatric test	MMSE WAIS-R SADS SRT (*Symptom Rating Test*)
Cuthill et al. (57)	Canada	Not specified	13 DM	Depression and anxiety	HAM-A (*Hamilton Anxiety Rating Scale*) HAM-D (*Hamilton Depression Scale*) SDS (*Zung Self Rating Scale*)
Douniol et al. (12)	France	Childhood form	28 DM1	Cognitive functions and psychological symptoms: hyperactivity, autism, depression, anxiety, alexithymia, impulsivity; sleepiness	WISC-R or WAIS the SAMUEL (visual–spatial construction abilities) MINI (*Mini-International Neuropsychiatric Interview*) the Dominic-R the whole ADHD section of the Diagnostic Interview Schedule for Children IV or Adult ADHD Self-Report Scale ASME (*Autism Mental Status Examination*) Children's Depression Inventory or BDI Spielberger State-Trait Anxiety Inventory Toronto Alexithymia Scale Lecendreux (for children) or Epworth (for adults) scales Eysenck Impulsivity self-report questionnaire
Echenne et al. (15)	France and Canada	Congenital, infantile/juvenile form	32 DM1	Longitudinal study over cognition	WAIS
Ekstrom et al. (58)	Sweden	Congenital and childhood form	57 DM1	Autism in childhood forms	ADI-R (*Autism Diagnostic Interview-Revised*) FTF (*The Five to Fifteen*) [to assess ADHD] SCQ (*Social Communication Questionnaire*) Griffiths Mental Development Scales, WAIS or WISC
Ekstrom et al., (14)	Sweden	Congenital and childhood form	55 DM1	Cognition and adaptive skills	Griffiths Mental Developmental Scale WPPSI-R or WISC-III or WAIS-III VABS (*Vineland Adaptive Behavior Scales*)
Franzese et al. (59)	Italy	Juvenile and adult-onsetform	28 DM1	Cognition and personality	Bender Visual Gestalt Test Wechsler-Bellevue Cancellation task MMPI Irritability-Depression-Anxiety scales of Snaith, Constantopoulos, Jardine, and McGuffin
Fujino et al. (7)	Japan	All except congenital form	60 DM1	Cognition and quality of life	MMSE WAIS-III VPTA (*Visual Perception Test for Agnosia*) [Story telling subtest] CAT (*Clinical Assessment for Attention*) WCST FAB TMT CAT Position Stroop test Fluency test Apathy Scale ESS PHQ9 (*Patient Health Questionnaire-9*) MFI (*Multidimensional Fatigue Inventory*) SRS (*Social Responsiveness Scale*) MDQoL (*Muscular Dystrophy QoL scale*)
Gallais et al. (22)	Canada	Adult or late-onset form	115 DM1	Cognitive decline in longitudinal study	MIRS WAIS-R CVLT (*California Verbal Learning Test*) RCFT Verbal Fluency BNT SCWT Ruff 2 and 7 Selective Attention Test
Gaul et al. (60)	Germany	Juvenile, adult and late-onset form	21 DM1, 21 DM2	Cognition in DM1 and DM2: depressive symptoms and fatigue; visuo-construction; attention; prefrontal functions	Profile of Mood States RCFT Symbol Digit Modalities Test VF (*Verbal fluency*) CAL (*Conditional-associative learning*) IGT (*Iowa Gambling Task*)
Goossens et al. (61)	Netherland-Belgium	Juvenile	24 DM1	Psychological characteristics in childhood	WISC-R CBCL
Jacobs et al. (62)	Belgium	Juvenile form	27 DM1	Cognitive functions and psychopathological	WAIS-III ASEBA (*Achenbach System of Empirically Based Assessment*) questionnaire about behavior checklist: YSR, ASR, CBCL and ABCL
Kalkman et al. (63)	Netherlands	Adult-onset form only	79 DM, 65 FSHD and 73 HMSN-I patients	Psychiatric disorders in neuromuscular disorders	SCID-I-R (*structured clinical interview for DSM-IV axis I*) BDI SCL-90 GHQ-12 (*General Health Questionnaire-12*) MRC CIS (*Checklist Individual Strength*)
Kleberg et al. (64)	Sweden	Juvenile, adult-onset and late-onset form	33 DM1, 30 healty controls	Cognitive functions and facial memory	RBMT-E (*Rivermead Behavioural Memory Test*) Neuropsychological tests: Block design test Vocabulary test RAVLT RCFT
Laberge et al. (65)	Canada	Adult-onset form	27 DM1	Sleepiness	ESS (*Epworth Sleepiness Scale*) DSS (*Daytime sleepiness Scale*) CFS (*Chalder Fatigue Scale*)
Malloy et al. (66)	USA	All forms	20 MMD, 20 normal control	Neuropsychological functions	WAIS-R WSCT WAB (*Western Aphasia Battery*) Benton's Facial Recognition Line orientation Three dimensional construction tests Wechsler Memory Scale [Russell revision]
Marchini et al. (67)	Italy	Not specified	24 DM1, 39 healty controls	Cognitive functions and other diseases related to DM	WAIS
Meola et al. (50)	Italy	Childhood, juvenile and Adult-onset form	19 DM2, 21 DM1	Cognitive functions and psychological characteristics	SCID (*Structured Clinical Interview*) MMSE Battery: TMT WCST TLT (*Tower of London Test*) ST (*Stroop test*) Lexical retrieval Computerized assessment of the multiple aspects of attentional performance
Modoni et al. (16)	Italy	Congenital and adult-onset forms	70 DM1 (10 congenital form, 60 Adult-onsetadult-onset)	Cognitive functions	Neuropsychological battery MMSE Subtest WAIS: RAVLT Rey-Osterrieth figure recall Digit Span Phonological and Semantic Word Fluency Raven Colored Progressive Matrices Temporal Rule Induction and SCWT TMT-B
Palmer et al. (68)	USA	All (except for congenital form)	21 DM1 (7 mMD and 14 pMD), 10 normal controls	Cognition and personality	MCMI (*Millon Clinical Multiaxial Inventory*) WAIS-R Wechsler Memory Scale RCFT Hooper Visual Organization Test Stroop A, B WSCT
Peric et al. (28)	Serbia	Childhood, juvenile, and adult-onset form	62 DM1	Personality	MIRS RSPM (*Raven's Standard Progressive Matrices*) MMCI SF-36 (*Short Form Health Survey*) [serbian version] INQoL
Perini et al. (69)	Italy	Adult-onset	17 DM1, 20 unrelated normal controls, 10 patients with SMA	Cognitive functions and psychiatric disorders	WAIS SADS (*Schedule for Affective Disorders and Schizophrenia*) MDRS (Muscular Disability Rating Scale) Event–Related Potentials
Prevost et al. (70)	Canada	All forms	308 subjects (44 carriers of DM1 gene and 264 non-carriers)	Psychological well-being after genetic test for DM	Questionnaire: Subjects were asked to complete items related to their social, economic and demographic background, the referring person to DNA testing, the reasons for testing and the recall of test result. PSI (*Psychiatric Symptom Index*)
Romeo et al. (71)	Italy	Not specified	50 DM1, 14 DM2, 44 healty subjects	Cognition and brain damage	MRI images MIRS, MRC scale Raven's progressive Matrices (PM47) Stroop (Word, Colour, Colour-Word) Fluency tests Wechsler Memory Scale CBT RCFT
Rubinsztein et al. (72)	UK	All except for congenital form	36 DM1	Memory and cognitive functions	Medical Research Council examination technique. NART (*National Adult Reading Test*) MMSE RBMT (*Rivermead Behavioural Memory Test*) WCST
Sansone et al. (17)	Italy	Not specified	56 DM1, 29 DM2 (follow-up: 20 DM1, 13 DM2)	Cognitive decline in longitudinal study	MRC scale MMSE RPM (*Raven's Progressive Colored matrices*) Token test Digit Span forward and Spatial Span Story Recall and Rey Recall RCFT TMT-A and B, TEA (*Alertness and Divided Attention*) TLT
Sansone et al. (73)	Italy	Not specified (probably congenital excluded) “age- and disease duration-matched, moderately-affected, ambulatory patients”	66 Italian patients with SMC, skeletal muscle channelopathies; 422 DM (DM1: 382; and DM2: 40) as control group	Quality of Life in neuromuscular disorders	INQoL SF-36 MMSE
Serra et al. (40)	Italy	Childhood and adult-onset forms	27 DM1, 16 healty controls	Cognitive functions and psychological characteristics; f-MRI	MMPI-2 neuropsychological battery fMRI
Serra et al. (39)	Italy	All forms (in particular adult-onset form)	20 DM1, 18 healty controls	Cognitive functions and Theory of Mind	MMSE MIRS WAIS-R RMET (*Reading the Mind in the Eyes*) ToMstory tests fMRI
Sistiaga et al. (27)	Spain	All except congenital form	121 DM1, 54 control subjects	Cognition and personality	MCMI-II WAIS-III MIRS ESS
Steyaert et al. (74)	Holland -Belgium	Congenital or juvenile form	16 DM1	Psychological symptoms and cognitive functions	WISC-R or WAIS CBCL (*Children's Behavior Checklist*) Child Depression Scale or BDI (*Beck Depression Inventory*) ADIKA (*Diagnostic Interview for Children and Adolescents*) [parent's interview]
Turnpenny et al. (75)	UK	All forms	55 DM1, 31 healty controls at risk	Cognitive functions	WAIS [short version]
Van Spaendonck et al. (76)	Netherlands	Early adult and adult MD	26 DM, 25 control subjects	Cognition	MDRS (*Muscular Disability Rating Scale*) MRC Zung depression scale WAIS CVLT (*California Verbal Learning Test*) Stroop test TMT B-A VF WCST
Winblad et al. (77)	Sweden	Adult-onset form	46 DM1, 31 healthy controls, 37 in contrast group	Psychological characteristics	TCI (*Temperament and Character Inventory*) [Swedish version]
Winblad et al. (18)	Sweden	Adult form (not mild)	47 DM1	Cognitive functions	WAIS-R FAS (verbal fluency) RCFT (*Rey Complex Figure Test*) SCWT-B (*Stroop Color and Word Test B*) RAVLT (*Rey Auditory Verbal Learning Test*) WCST (*Wisconsin Card Sorting Test*) TMT-A TMT-B (*Trail Making Test A and B forms*)
Winblad et al. (78)	Sweden	Adult-onset form	50 DM1, 41 healty controls	Psychological characteristics and facial emotion recognition	POFA (*Ekman and Friesen's Pictures of Facial Affect*) WAIS-R RAVLT RCFT COWAT (*Controlled Oral Word Test*); WCST CWT (Color Word Test) TMT TCI
Winblad et al. (21)	Sweden	Adult-onset form	31 DM1, 47 subjects in a clinical contrast group	Psychological symptoms (depression) and cognitive functions	BDI (*Beck Depression Inventory*) [Swedish version] MIRS tests: Vocabulary FAS Visual construction RCFT Block design Picture completion RAVLT TMT Digit symbol Digit span Spatial span SCWT WCST
Winblad et al. (23)	Sweden	Adult-omset form	37 DM1	Cognitive functions at follow-up	MIRS FAS Arithmetic Block design RCFT (*Rey Complex Figure Test*) RAVLT TMT-A, TMT-B Digit Span Spatial span SCWT-B WCST

Our research contains an original protocol that investigates both cognitive and psychopathological domains. The selection of instruments was based on the review of the current available literature which was previously described (see Table 1). Our study focused on the neuropsychological and psychological aspects of DM1 population. Most of the previous studies focused on cognitive functioning and its association with the medical characteristics of the disease, such as disease form or CTG length (12, 14–16, 18, 22, 23, 27, 30, 40, 50, 53, 58–60, 64, 67–69, 71, 72, 74, 75, 78) and other studies looked at the psychological characteristics (7, 12, 21, 26–28, 40, 50, 52–54, 56–63, 68–70, 73, 74, 77, 78). The purpose of this research was to investigate both cognition and a psychological symptomatology association. We opted for a battery containing screening tests (MMSE, for a general cognitive screening; FAB for the assessment of the executive functions) and we decided to go for ENB-2 battery vs. WAIS, because it is validated on the Italian population and it offers the advantage of a shorter assessment time (7, 11, 12, 14, 15, 18, 22, 26, 27, 30, 35, 39, 51, 53, 56, 58, 61, 62, 66–69, 74, 76, 78).

For the assessment of the psychological characteristics, we adopted the SCL-90-R, based on the Canadian study of Bertrand et al. (26); since this test allows to inquire nine psychological dimensions and not only depression or anxiety that are used in many other studies (21, 60, 57, 54, 52).

The Clinical Psychology Service of the IRCCS Policlinico San Donato was activated in August 2016 and successively a collaboration with the Neurology Unit was established. A neuropsychological and psychological assessment research protocol on DM1 patients was reviewed and approved on the 24th of October by the ethical committee Ospedale San Raffaele in Milan, Italy (registration number 135/INT/2017) and was conducted according to the principles expressed in the Declaration of Helsinki, the institutional regulation and Italian laws and guidelines. Written informed consents were obtained from the patients.

### Sample characteristics

In our study, 31 DM1 patients referred to the Neuromuscular Center of the IRCCS Policlinico San Donato and registered in the Italian Registry for “Myotonic Dystrophy Type 1 and Type 2” were enrolled.

The following inclusion criteria were utilized: (i) age equal to or above 18 years; (ii) patients able to comprehend the conditions of the study and to participate to the entire duration of the study. Congenital and infantile form, pregnant patients or women who are breastfeeding and patients with IQ ≤ 91 were excluded. Only patients with IQ in the norm or above (IQ 92–109 normal/110–126 > normal) were enrolled in order for them to be able to complete the whole neuropsychological and psychological assessment.

Patients were subdivided according to the five clinical forms based upon the age of onset as recently proposed by De Antonio et al. (6): (i) congenital form (onset from birth to 1 month); (ii) infantile form (onset from >1 month to 10 years); (iii) juvenile form (onset from 11 to 20 years); (iv) adult form (onset from 21 to 40); v) late onset form (onset after the age of 40 years).

Patients were categorized according to CTG expansion range: E1 (51–150), E2 (151–500), E3 (501–1,000), E4 (>1,000) (16).

Muscular impairment was assessed using: (i) Muscular Impairment Rate Scale (MIRS) between 1 and 5: 1 representing no muscular impairment and 5 representing severe muscular weakness; (ii) Medical Research Council Scale (MRC), a standard muscle power test, which grades on a scale of 0–5 in relation to the maximum expected for that muscle.

We measured individual daytime sleepiness with the Epworth Sleepiness Scale (ESS), a self-reported questionnaire that asks the patient to score (from zero to three) their likelihood of falling asleep in eight routine life situations. A final score ranging from 0 to 9 is considered normal; a score from 11 to 15 is typical of mild to moderate sleep apnea; a score of 16 and above is associated with severe sleep apnea.

### Methods

The design of the study is evaluative, observational and prospective.

The assessment process was conducted by a neuropsychologist of the Clinical Psychology Service of the IRCCS Policlinico San Donato. The protocol administration lasted for an average of 90/110 min and included the following steps:

- Clinical and Neuropsychological Interview
- Neuropsychological assessment
- Psychological assessment

### Clinical and neuropsychological interview

The purpose of the interview was to assess the following aspects of patients: personal history (educational, family, occupational, cognitive, social, medical, psychological), cognitive disorders, motivation and apathy, emotion and emotional disorders, self-control, sense of reality and psychotic type disorders, premorbid personality, personality modification, disease awareness, impact of disease on quality of life, family relationships and therapeutic alliance.

### Global cognitive functioning assessment

The Global Cognitive Functional Assessment was carried out through the administration of the Mini Mental State Examination (MMSE) (79).

This is a test for the assessment of intellectual and efficiency disorders and the presence of intellectual impairment. It consists of thirty items that refer to seven different cognitive areas: orientation in time, orientation in space, recording of words, attention and calculation, memory, language, constructive praxia. The total score is between a minimum of 0 and a maximum of 30 points. A score equal to or <18 indicates a serious impairment of cognitive skills; a score between 18 and 23 indicates a moderate to mild impairment, a score of 25 is considered borderline.

### Executive functioning assessment

The Executive Functioning Assessment was carried out through the administration of the Frontal Assessment Battery (F.A.B.) (80).

It is a test for the assessment of the efficiency of executive functions and analyzes the following cognitive abilities: conceptualization, shifting/switching, planning, inhibitory control, sensitivity to interference, environmental autonomy (81). The cut-off limit for the adjusted score is set at 13.50.

### Cognitive domains assessment through ENB-2 battery (italian version)

The ENB-2 assessment battery offers a qualitative and quantitative analysis of patient performance and is validated on the Italian population from 15 to 96 years and has the advantage of offering a shorter assessment time of cognitive domains.

The battery consists of 16 tests that analyze the following cognitive areas (82): short, long term and working memory, integration capacity, visual-spatial research, divided attention and attention shifting/switching, psychomotor speed, verbal comprehension, lexical recovery, logical and abstract reasoning, critical sense, capacity for discrimination, complex copy skill, praxis, and mental representation.

### Psychological evaluation through symptom check list 90-R (SCL-90-R)

Similarily to Bertrand et al. (26), we selected the SCL-90-R questionnaire. It is a self-assessment questionnaire built to provide a standardized measure of an individual's current psychological and/or psychopathological status over to past week, applicable to normal or psychiatric populations of adults and adolescents (83). So far there is no evidence of other Italian studies on patients diagnosed with DM1 where a cross relation between Neuropsychological tests and SCL-90-R were performed. The objective in our study was to assess other psychopathological categories other than anxiety, depression, irritability and avoidant personality, and with the SCL-90-R there is also the possibility to take into consideration the 3 symptomatological indexes described hereunder. The nine primary dimensions investigated are: Somatization, Obsessive-Compulsive Dimension, Interpersonal Sensitivity, Depression, Anxiety, Hostility, Phobic anxiety, Paranoid ideation and Psychoticism.

The clinical information that can be obtained from the administration of SCL-90-R are also summarized into three indexes: Global Severity Index (GSI), Index of Disorder of Positive Symptoms (PST) and the Total Index of Positive Symptoms (PSDI)

The study of the psychometric properties of the SCL-90-R has provided satisfactory results for what concerns the internal coherence of the instrument and its reliability test–retest. Further studies confirmed the validity of construct and the predictive validity (Italian adaptation by E. Preti, A. Prunas, I. Sarno and F. Madeddu) (84).

Mean (and SDs) or median (and 1st and 3rd quartiles) as more appropriated, were used to describe continuous variables, while counts and percentage were used for categorical variables. The normality assumption was checked with the use of the Shapiro–Wilk test. Continuous parameter of diseases duration were analyzed using the two-sample Wilcoxon test for non-normally distributed data. Significance level was set at *P* < 0.05.

The SAS software, version 9.4 (SAS Institute, Inc., Cary, NC) was used for the analysis.

## Results

Sociodemographic, clinical, and psychological characteristics of DM1 patients are reported in Table 2.

**Table 2:** Sociodemographic, clinical, and psychological characteristics of DM1 patients.

	**All DM1 (*n* = 31)**	**Juvenile DM1 (*n* = 18)**	**Adult-onset DM1 (*n* = 9)**	**Late-onset DM1 (*n* = 4)**
Age (years)	37.0 (25.0–50.0)	29.5 (24.0–40.0)	39.0 (36.0–52.0)	55.0 (48.0–58.5)
Gender (% Male)	61	55.6	55.6	100
Age at onset (*n*)	20.0 (16.0–25.0)*	16.0 (12.0–20.0)	31.0 (25.0–33.0)	43.0 (41.0–45.0)*
Disease duration (years)	14.0 (6.0–24.0)	15.0 (6.0–24.0)	14.0 (5.0–18.0)	8.0 (2.0–14.0)
**CTG REPEAT RANGE (*****n*****)**				
E1 (51–150)	2 (6.5%)	0	0	2 (50.0%)
E2 (151–500)	16 (51.6%)	10 (55.6%)	5 (55.6%)	1 (25.0%)
E3 (501–800)	6 (19.4%)	5 (27.8%)	1 (11.1%)	0
E4 (>800)	3 (9.7%)	2 (11.1%)	1 (11.1%)	0
not determined	4 (12.9%)	1 (5.6%)	2 (22.2%)	1 (25.0%)
MIRS	3.0 (2.0–4.0)	3.0 (2.0–4.0)	4.0 (3.0–4.0)	2.0 (1.0–3.5)
MRC	125.0 (112.0–127.7)	125.0 (111.0–128.0)	122.7 (115.0–125.0)	126.0 (112.8–130.0)
Epworth Sleepiness Scale	6.0 (4.0–9.0)	7.0 (6.0–12.0)	6.0 (4.0–7.0)	5.5 (3.0–6.0)
Education (years)	13.0 (8.0–13.0)	13.0 (13.0–13.0)	13.0 (8.0–16.0)	8.0 (8.0–8.0)
% employed	71.0	72.2	66.7	75
% in a relationship	45.0	22.2	66.7	100
**ITALIAN REGION**				
North	24 (77.4%)	12 (66.7%)	8 (88.9%)	4 (100%)
Center	3 (9.7%)	3 (16.7%)	0	0
South	4 (12.9%)	3 (16.7%)	1 (11.1%)	0
**MMSE**				
% in the norm range	96.8	94.4	100	100
**ENB-2**				
% in the norm range	80.6	72.2	88.9	100
**FAB**				
% in the norm range	71.0	61.1	77.8	100
% upper limits of the norm	16.1	22.2	11.1	0
% lower limits of the norm	9.7	16.7	0	0
% deficit	3.2	0	11.1	0
**SCL-90-R**				
% T-scores in the normal range
Global severity index	80.6	83.3	77.8	75.0
Positive symptom total	87.1	88.9	77.8	100
Positive symptom distress index	83.9	88.9	77.8	75.0
Somatization	83.9	83.3	77.8	100
Obsessive-compulsive	90.3	94.4	77.8	100
Interpersonal sensitivity	80.6	83.3	77.8	75.0
Depression	80.6	88.9	66.7	75.0
Anxiety	96.8	100	88.9	100
Hostility	77.4	83.3	55.6	100
Phobic anxiety	90.3	94.4	77.8	100
Paranoid ideation	83.9	83.3	77.8	100
Psychoticism	83.9	88.9	77.8	75.0

* *Patients excluded since asymptomatic. Median value (interquartile range)*.

A total of 31 patients with DM1 (19 men and 12 women) participated in this study. All patients were Italian and 77.4% were from northern Italy. Patients were classified as having juvenile (58.1%), adult-onset form (29.0%) and late onset (12.9%) forms of the disease.

The global cognitive functions screened through the MMSE were normal in the 96.8% of the entire DM1, only one patient reported a severe impairment in the spatial and temporal orientation, memory, language, praxis, attention and calculation.

Concerning the different specific cognitive domains, assessed through the ENB-2, it was shown that 80.6% of patients were in the range of normality and 19.4% were below the norm. The most negatively affected domains were: attention (25.8%), mental representation (29.0%), praxis (32.3%) and discrimination (22.6%).

The assessment of executive functions through the Frontal Assessment Battery (FAB) showed that 77.8% of patients had a normal frontal functioning and 22.2% had an impairment.

About one fifth of our DM1 patients showed SCL-90-R scores in the range of “normal” and the same distribution also occurred among the different DM1 forms.

19.4% of patients showed a moderate-high level of symptoms intensity index (GSI), 12.9% reported a moderate number of symptoms (PST) and 16.1% reported a moderate-high intensity level of the perceived symptoms (PSDI).

In relation to the nine symptomatic dimensions of SCL-90-R, 16.1% of patients refer moderate-high level of somatization (SOM), this also emerged during the clinical interview where patients often reported a strong feeling of uneasiness with their own body. Three patients (9.7%) showed obsessive-compulsive symptoms (O-C), others (9.7%) reported phobic anxiety (PHOB) and only one patient suffered from anxiety (ANX). 19.4% of the sample had a depressive state (DEP), this percentage is higher in comparison to the prevalence of depression in the normal population that stands around 2–5% (85). 19.4% of patients reported a high level of interpersonal sensitivity (IS), a typical psychological trait of people who experience feelings of inadequacy and inferiority with respect to others. Furthermore, the presence of hostility feelings (HOS in the 22.6%) and paranoid ideation (PAR in the 16.1%) can be seen as an indicator of existing relational problems. 16.1% of the sample reported the presence of psychotic symptoms (PSY) like social isolation and negative symptoms of schizophrenia.

It has been identified that the disease duration has a clinical impact on the cognitive performances assessed through the ENB battery. Patients who obtained results in the normal range of the ENB have a disease duration median of 6 years (5.0–22.0) vs. 23.5 (18.0–34.0) for those who obtained results below the norm (*P* = 0.03).

No statistical significance was found for other sample characteristics, such as % in a relationship, % employed and Italian region in correlation with ENB (respectively *P* = 0.66; *P* = 0.18; *P* = 0.11).

This might be due to the small sample size.

### Illustrative case study

In this paragraph we illustrate a clinical case report of a family of four people (three diagnosed DM1 and one not affected by disease): Father, 53 years old, DM1 late onset form; Mother, 50 years old, not affected; Son 1, 25 years old, DM1 juvenile form; Son 2, 21 years old, DM1 juvenile form (neuropsychological and psychological data collected during the first visit are available in Table 3).

**Table 3:** A neuropsychological and psychological assessment of a family.

	**Cognitive functions**			Psychological dimensions (SCL-90-R)*											
	**MMSE**	**ENB-2**	**FAB**	**GSI**	**PST**	**PSDI**	**SOM**	**O-C**	**I-S**	**DEP**	**ANX**	**HOS**	**PHOB**	**PAR**	**PSY**
Father	30/30	86	18/18	45	46	45	40	48	40	46	51	46	45	44	46
Son 1	30/30	85	18/18	76	58	88	56	84	64	75	56	60	43	84	85
Son 2	30/30	90	18/18	45	45	47	56	45	42	44	42	48	48	46	42

* *T-score : 25–55 (absence/normal), 55–65 (moderate), 65 > (high)*.

This family was selected in order to test a family-centered psychosocial care program since the family members have always been involved in the Italian Myotonic Foundation activities, as well as actively promoting initiatives around the DM1 disease.

Since this family is representing most of the disease forms, it might be representative of the various ages at disease onset and the different neuropsychological and psychological functioning as well as providing examples of different coping capabilities.

We designed a family-centered psychosocial care program in order to support the well-being of the different members. The main needs were different and were inferred from the SCL-90-R results and what emerged from the first clinical interview. The main objective of family-centered psychosocial care program was to strengthen the emotional resilience of chronically ill patients and their families. It aims to enhance a “holistic” treatment of the patient and the family as “a whole”—that is, from a body and mind perspective—and the psychosocial development of the patient and his or her family (86).

We met the father twice in order to assess his needs for support based on the fact that his two sons have also been diagnosed DM1. It immediately appeared that he had a good awareness of the disease and its implications as well as a strong resiliency. This also can be seen in Table 3, where all psychological dimensions are in the range of absence or normal. His demand for support was about taking care of his two sons and his wife who was feeling guilty because she was the only one not affected by the disease.

Son 1 is the most serious case in the family. From the very first clinical interview a clear picture of psychological pain and isolation from any social relationship emerged. The patient had just been left by his girlfriend since she was unable to cope with his disease and did not see any possible future in their relationship. He participated to 4 monthly psychotherapy sessions, in which the topics were the elaboration of the breakup and disease acceptance. The patient had no cognitive impairment but scored highly on the following psychopathological traits; O-C, DEP, PAR, PSY with also a high Global Severity index.

Son 2 demonstrated a high intelligence, with very quick reaction times and a very positive outlook on life. He only reported that he felt a bit excluded in the family since everyone was focused on Son 1 for his serious health conditions. He participated to five psychological sessions and during the clinical interviews it emerged that he was basically neglecting the fact the he was diagnosed DM1. There was a focus on the creation of awareness by using his strong self-perception as a solid point to be exploited. In fact, he achieved the best results in the cognitive performance and did not show any psychopathological traits.

The mother reported a strong sense of guilt for being healthy and a strong fear of losing her children. In order to protect them she was not allowing herself to feel any emotion since she wanted to appear strong and protect them from everything. In the psychological sessions she was allowed to express her feelings and to work on the acceptance of the situation.

The following indications were given at an individual level when it comes to the family dynamics:

- Openly sharing information, emotions and fears In order to reduce the stress and anxiety level related to DM1, it was important to provide a clear disease perspective to the patients and family members in collaboration with the neurologist. By doing so there was also a verification of the disease awareness among the different family members through the psychotherapy sessions;
- Being realistic and coping with what the daily life was presenting them. It was important to work on a program to enhance a normal flow of life and step out from a pathological mode, by living in the here and now;
- Respecting the weaknesses of each other in order to provide mutual support;
- Psychological support once a month to monitor the situation by offering a dedicated individual session in order to enable the patients and their family members to share their fears, emotions and concerns.

Table 3 indicates the neuropsychological and psychological results of the family where three members have been diagnosed with DM1.

The data shown in Table 3 are referring to the neuropsychological and psychological dimensions assessed during the first visit of the family members. A follow up assessment will take place at the end of the psychotherapy sessions by the end of 2018.

From a clinical observation perspective, we noticed an improvement of the general mood of son 1 who has developed a social network, has started socializing and has also started a part-time job showing an increased awareness and new abilities to cope with his disease. The improvement of the clinical symptoms of son 1 is having positive effects on the whole family with reduced stress for all members and a more supportive family environment can now be observed. Follow up data will be available at the end of the year.

## Discussions and conclusions

Recently in our hospital, the Clinical Psychology Unit of the IRCCS Policlinico San Donato in collaboration with the Neurology Unit has developed a battery of tests to evaluate cognitive assessment, personality traits and depression based on the results obtained by the Canadian team (26). A small group of DM1 patients has been tested with the intent to give them the possibility to access to psychological support other than to standard clinical care.

Regarding the main objective of this study, our results show relevant personality impairment in DM1 patients, mainly hostility, depression and interpersonal sensitivity. These symptomatic dimensions could be also explained with low empathy and low collaboration that Winblad measured with the Temperament and Character Inventory (TCI) (87).

The question which still remains open in our cohort is if these psychopathological traits were evident before or after the DM1 was diagnosed (88).

In our sample, cognitive impaired DM1 patients have a general tendency to maximize or minimize their positive symptoms and as shown in the literature, our DM1 patients tend to have a deficit in relations due to a low social cognition functioning (ToM, Theory of Mind) (38).

When it comes to the neuropsychological aspects, we have noticed that most negatively affected cognitive domains were praxis, mental representation, attention and discrimination.

Both neuropsychological and psychological impairments might have an impact on the patients' perception of quality of life. In fact, both quality of life and the patients' actual health, can be influenced by two main factors; decisions made by physicians and the patient's compliance. When a health condition is particularly serious, decisions regarding a diagnosis or treatment can often make a difference between life and death. For this reason, it is relevant for physicians to be able to make the best possible decisions and that the patients can understand what their clinical situation is and to follow the medical recommendations given (46).

In line with with our additional objective the emerging clinical implications and available data can be used as a basis to provide neurocognitive rehabilitation programs as well as psychological support program to DM1 patients and their families. Our study shows a bigger percentage of cognitive impairment when it comes to the praxis, mental representation, attention and discrimination domains, for which we are designing group and individual rehabilitation programs focused at the improvement of these functioning. The program will be available as soon as the research is completed and a bigger sample is available.

Since the study started last November 2017 and due to the small current sample size, we decided to describe the population without other inferential statistical analysis, which will be integrated in a later phase of the study when more data will be available.

At the moment we cannot envisage any effective medical treatment for DM1 patients and the data available in this research could effectively be used to work on DM1 patients' empowerment by making use of their cognitive reserve and stimulate through proper neuro-rehabilitation programs the main affected cognitive domains and offer psychological support program. The rehabilitation protocol will focus on attention, memory, praxis executive functions while on the psychological side enhance the disease awareness and patients' coping strategies.

Our transversal study confirms the results of the longitudinal trial of Winblad et al. (23), who highlighted a cognitive deterioration at follow up time hence confirming the relation between the disease duration and cognitive performances.

This confirms the core importance of designing an effective rehabilitation protocol at the end of the data collection.

In these days, patients spontaneously search for information on the internet, and this might help them get accurate information but it may also lead them to getting lost on the web, getting information from unknown sources and inaccurate information. In order to get an effective involvement of patients in their care and their increased engagement, it is essential to enact strategies to improve their health literacy (45). It has been observed in the medical settings that usually patients require more information than they are given from health professionals, and they feel that they would like to be more involved in therapeutic procedure (44).

This is very much aligned to the innovative P5 approach, which represents the psycho-cognitive aspects to be considered in order to empower the patient, increase quality of life and transform them from a passive recipient into an active participants in the treatment process (46).

Because of the inherited nature of DM1, a diagnosis in one individual has implications for other family members, hence the care system is addressed to the family as a whole. Although the goal is clear, there are many challenges to proceed in this direction and it is necessary to create occasions where the patients have the possibility to be empowered. This means putting the patients in a condition that they feel they have control on their disease which in turn leads to an improvement in therapeutic outcomes (45).

In conclusion, in line with previous studies, there are indications that also the Italian patients have a high probability of developing psychiatric disorders. More data are necessary in order to make inferences about the relationships between psychosocial, neuropsychological and medical variables. Studies about the efficacy of interventions specifically established for these patients and their family members are necessary.

The current limitation is the effect of the small sample size. The complete analysis with the evaluation of primary and secondary end points of this study will be available when data collection is finished.

## Author contributions

EC proposed the study design and the idea, analyzed the literature, its development and gave the final approval of the manuscript. EGB evaluated the neuropsychological and psychological assessment of patients, contributed to the development and the revision of the work and, agreed to the approval of the manuscript. MB executed the statistical analysis and contributed to the development and revision of the work and, the agreed to the approval of the manuscript. RC executed the histopathological and the molecular biology analysis and, contributed to the development and revision of the work and, agreed to the approval of the manuscript. BF provided the clinical data and contributed to the development and revision of the work and, agreed to the approval of the manuscript. EB provided the clinical data and contributed to the development and revision of the work and, agreed to the approval of the paper. GM organized the meeting, contacted the patients and supervised the medical aspects of the patients. GB also contributed to the development and the revision of the work and agreed to the approval of the manuscript. SB made substantial contributions to the analysis and the interpretation of the data in the manuscript and, revised it substantially. SB approved the final submitted version.

### Conflict of interest statement

The authors declare that the research was conducted in the absence of any commercial or financial relationships that could be construed as a potential conflict of interest.

## Footnotes

^1^During the OMMYD-1 Workshop, four neuropsychological tests have been found as relevant in a clinical trial. These tests are Trail Making Test A and B, Stroop Color and Word Test, FAS (oral word association), and WAIS-R Block Design subtest. In our ENB-2 battery, similar tests were used to challenge the same cognitive areas.
