# Supplementary material for: Neuropsychological and Psychological Functioning Aspects in Myotonic Dystrophy Type 1 Patients in Italy
Source: Front Neurol. 2018 Sep 19;9:751. doi: 10.3389/fneur.2018.00751 (PMC6160752; doi:10.3389/fneur.2018.00751)
Supplement: Supplementary file 1 [file Data_Sheet_1.docx]

**Appendix 1**

A review of the literature was conducted, and the following key words were utilized; “myotonic dystrophy” AND “psych*”. All studies conducted in a clinical sample of DM patients that investigated neuropsychological, psychological or psychiatric aspects were included. The research of the literature was conducted on the PubMed and PsycINFO databases. The authors followed guidelines for systematic reviews formulated by Perestelo-Pérez, L. (2013). Standards on how to develop and report systematic reviews in Psychology and Health. *International Journal of Clinical and Health Psychology*, *13*(1), 49-57. In accordance with the objectives of the review, the authors have focalized on studies that inquire psychological dimensions.

The research was conducted in November 2017 for all studies available until 30/11/2017. 97 studies were considered as a result of this review on the basis of these inclusion criteria: keywords, databases and time of research. After reading the selected articles, other relevant articles cited in the these studies which were not previously included were also added. Exclusion criteria were: non-English language studies and when the title or abstract was not pertinent to the focus of this study.

Finally 46 relevant articles were identified, presented in Table 1.
